# Current approaches and advances in the imaging of stroke

**DOI:** 10.1242/dmm.048785

**Published:** 2021-12-07

**Authors:** Pragati Kakkar, Tarun Kakkar, Tufail Patankar, Sikha Saha

**Affiliations:** 1 Leeds Institute of Cardiovascular and Metabolic Medicine, Faculty of Medicine and Health, University of Leeds, Leeds LS2 9JT, UK; 2 Leeds Teaching Hospitals NHS Trust, Leeds LS9 7TF, UK

**Keywords:** Stroke, Ischaemic stroke, Haemorrhagic stroke, Neuroimaging, Computed tomography, Magnetic resonance imaging

## Abstract

A stroke occurs when the blood flow to the brain is suddenly interrupted, depriving brain cells of oxygen and glucose and leading to further cell death. Neuroimaging techniques, such as computed tomography and magnetic resonance imaging, have greatly improved our ability to visualise brain structures and are routinely used to diagnose the affected vascular region of a stroke patient's brain and to inform decisions about clinical care. Currently, these multimodal imaging techniques are the backbone of the clinical management of stroke patients and have immensely improved our ability to visualise brain structures. Here, we review recent developments in the field of neuroimaging and discuss how different imaging techniques are used in the diagnosis, prognosis and treatment of stroke.

## Abstract (precis)

**Summary:** Stroke imaging has undergone seismic shifts in the past decade. Although magnetic resonance imaging (MRI) is superior to computed tomography in providing vital information, further research on MRI is still required to bring its full potential into clinical practice.

## Introduction

Strokes are the second most frequent cause of death after myocardial infarction and the leading cause of long-term disability worldwide (O'Donnell et al., 2016). According to the Global Burden of Disease Study 2015, estimated neurological disorders, including stroke, account for 16.8% of all deaths (Katan and Luft, 2018; Feigin et al., 2017). The risk of stroke generally increases in people over 60 years as blood vessels become harder and narrower with age.

There are two main categories of stroke – ischaemic and haemorrhagic. Ischaemic stroke begins when a blood clot causes a sudden cessation of blood supply to a part of the brain, while haemorrhagic stroke is caused by rupture of blood vessels in or around the brain (Box 1). Both types of strokes can cause death or permanent disability, with symptoms that can include loss of coordination and strength in limbs, numbness, loss of vision, facial palsy and speech abnormalities. In the past few decades, 7.2 million incidences of ischaemic stroke and 3.2 million incidences of haemorrhagic stroke have been reported globally in people aged 20-64 years. Mortality from stroke is rising in developing countries while reducing in developed countries, particularly among young adults (Krishnamurthi et al., 2015).

**Box. 1. Ischaemic and haemorrhagic stroke**

There are two categories of ischaemic stroke – thrombotic stroke and embolic stroke. A thrombotic stroke occurs when a thrombus (blood clot) forms within a blood vessel in the brain, while an embolic stroke occurs when a blood clot travels from the body to blood vessels in the brain (Caplan, 2006). According to the American Heart Association, ischaemic stroke accounts for ∼87% of the total stroke cases in the USA, while 10% of stroke cases are intracerebral haemorrhage and 3% subarachnoid haemorrhage (Caplan, 2006; Mozaffarian et al., 2016). According to a survey of 84,184 patients admitted with stroke in England, Wales and Northern Ireland, 87.1% had been diagnosed with acute ischaemic stroke and 12.2% with haemorrhagic stroke (Xu et al., 2018). Most ischaemic stroke patients have occlusions in the distal internal carotid artery, proximal middle cerebral artery or basilar artery, and occlusions in these major cerebral arteries produce acute neurological deficits if not treated urgently (González, 2012). Several factors contribute to ischaemic strokes, including age, atherosclerosis, hypertension, smoking, high cholesterol and diabetes mellitus (Gaillard, 2013). Haemorrhagic stroke is caused by the rupturing of blood vessels in or around the brain. This type of stroke is classified into four categories based on where in the brain the vessel rupturing takes place. The four categories are intracerebral, subarachnoid, subdural and epidural haemorrhage. Multiple factors such as trauma, head injury, hypertension, aneurysm and anticoagulant medication contribute to this type of stroke (Caplan, 2006; Unnithan and Mehta, 2021).

Apart from acute or permanent physical disability as a post-stroke outcome, stroke also has considerable socio-economic impacts. Based on a report published by the American Heart and Stroke Association, stroke accounts for 1.7% of national health expenditure in the USA (Ovbiagele et al., 2013). This report also projects that the total direct and indirect cost of stroke will increase from $105.2 billion in 2012 to $240.7 billion in 2030. Based on UK statistics, the economic cost associated with acute ischaemic stroke accounts for 3-5% of the total annual health expenditure (Xu et al., 2018). The annual direct and indirect cost of stroke is £25.6 billion, with an estimated 1 million stroke survivors in the UK (King et al., 2020). Thirteen per cent of these costs are to the National Health Service (NHS), 20% derive from social care costs, 61% from unpaid care costs and 6% from reduced productivity. The clinical management costs of stroke patients include diagnosis using neuroimaging techniques, blood tests and the required interventions.

The clinical management of stroke patients is guided by the concept of ‘time is brain’. This concept emphasises the importance of providing urgent medical treatment to stroke patients (González, 2012), which is mainly informed by neuroimaging. Rowley (2001) coined the term ‘Four P's’ – for parenchyma, pipes, perfusion and penumbra – to describe the aim of stroke imaging (Rudkin et al., 2018; Rowley, 2001). According to the UK National Institute for Health Care and Excellence (NICE) guidelines, computed tomography (CT) is the first line of diagnosis of hyperacute stroke [National Guideline Centre (UK), 2019]. This includes the use of non-contrast CT (NCCT), CT angiography (CTA) and CT perfusion (CTP) (Table 1) to identify thrombectomy candidates and to distinguish between ischaemic and haemorrhagic stroke. By contrast, magnetic resonance (MR) imaging (MRI) is used mainly for its ability to generate precise and high-resolution anatomical images of the brain. MRI-based techniques, such as MR angiography (MRA; see Glossary, Box 2) and diffusion-weighted imaging (DWI; Box 2), can provide more detailed information about a clot's size and location, and generate a clear picture of acute infarction (Kidwell and Wintermark, 2010; Mortimer et al., 2013; Lee and Demchuk, 2015; Vo et al., 2015). The nuclear imaging technique, positron emission tomography (PET), is also being researched to assess its utility for stroke diagnosis (Dundar et al., 2019).

**Box. 2. Glossary**

**Alberta Stroke Program Early Computed Tomography Score (ASPECTS):** a widely used, 10-point computed tomography scan score used to assess ischaemic changes in the brains of patients with middle cerebral artery stroke. For every region affected, 1 point is deducted from an initial score of 10.

**Chemical exchange-dependent saturation transfer (CEST):** a measure of chemical exchange between water protons and neighbouring molecules with exchangeable protons. It is a contrast-enhancement technique used to detect a specific molecule with free protons by applying a radiofrequency corresponding to that molecule.

**Diffusion-weighted imaging (DWI):** this magnetic resonance imaging (MRI) sequence detects the movement of water molecules. In ischaemic brain tissue, water movement becomes restricted intracellularly, which appears as a bright spot or area on the image.

**DWI/PWI mismatch:** the volume difference between a lesion visualised on a diffusion-weighted image (DWI) and on a perfusion-weighted image (PWI), which gives an estimate of the infarct core and penumbra.

**Endovascular therapy (EVT):** a mechanical procedure (also known as a thrombectomy) performed to remove blood clots, in which microcatheters are inserted into a blood clot via the arm or groin.

**Fluid attenuation inversion recovery (FLAIR) sequence:** an MRI sequence similar to the T2-weighted sequence except that the cerebrospinal fluid will appear dark and abnormalities will remain bright.

**Gradient recalled echo (GRE) sequence:** an MRI sequence also known as a T2*-weighted sequence. It can detect the smallest alterations in the magnetic field and improves the rate of lesion diagnosis.

**Haemorrhagic transformation:** a complication in cerebral ischaemic stroke patients. It occurs when peripheral blood flows from the disrupted blood-brain barrier into the brain.

**Infarct core:** region of the brain that receives no blood when a blood vessel is occluded.

**MRI sequence:** the pattern of radiofrequency pulses and the alteration of magnetic fields to generate an image that details a specific region of interest.

**Magnetic resonance angiography (MRA):** an MRI sequence that is specifically used for the evaluation of blood vessels.

**Penumbra:** region of the brain around the infarct core that receives low blood flow and is at risk of developing an infarction. It is potentially salvageable with appropriate medical intervention.

**Perfusion-weighted imaging (PWI):** an MRI sequence that measures the cerebral perfusion through the assessment of haemodynamic parameters such as cerebral blood flow, cerebral blood volume and mean transit time.

**Radiofrequency (RF) field shimming:** a process to correct an inhomogeneity in the magnetic field at high magnetic field strength (>7 Tesla).

**Recombinant tissue plasminogen activator (rtPA):** an intravenous drug that activates the conversion of plasminogen to plasmin, an enzyme responsible for dissolving blood clots. The process of dissolving clots is known as thrombolysis.

**Reperfusion therapy:** medical treatment performed using drugs or surgery to restore blood flow through blocked blood vessels.

**Specific absorption rate (SAR):** rate at which the electromagnetic energy of radiofrequency pulses is absorbed by biological tissues during an MR scan. Increased SAR can damage the tissues.

**Stroke mimic:** a medical condition with similar clinical symptoms to a stroke but caused by a non-vascular disorder.

**Susceptibility-weighted imaging (SWI):** an MRI sequence similar to the GRE sequence but more sensitive than GRE in detecting haemorrhages.

**T1-, T2-weighted sequences:** the basic MRI sequences that depict the differences in longitudinal (T1) and transverse (T2) relaxation times of tissues under diagnosis.

**Tesla:** SI unit of the magnetic field.

**Thromboembolism:** obstruction in the blood flow through a blood vessel caused by a clot/thrombus.

**Table 1.**
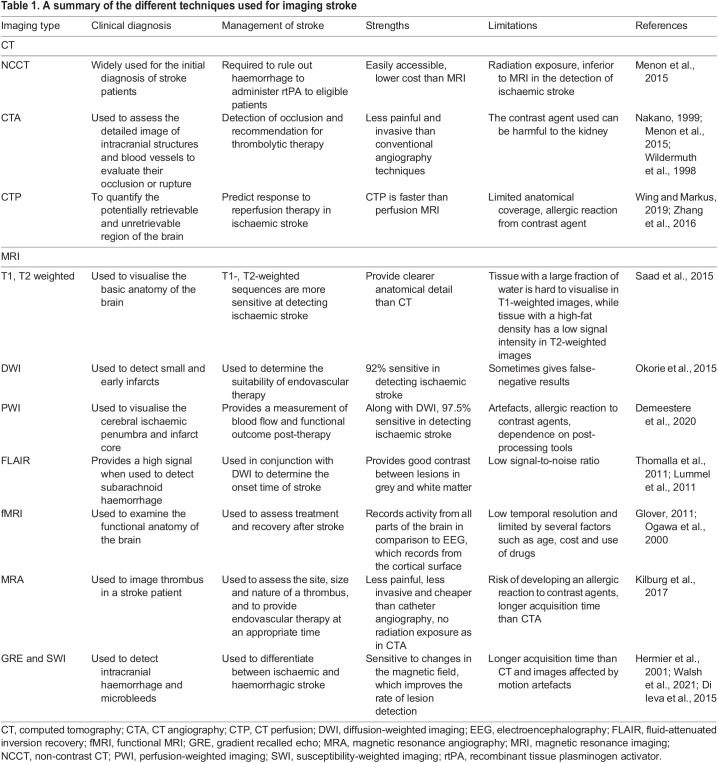
A summary of the different techniques used for imaging stroke

In this Review, we discuss recent advances in imaging technologies, their uses, and impact on the early diagnosis and treatment of stroke. We also discuss recent, significant developments in neuroimaging, such as artificial intelligence and machine learning, imaging with contrast agents, thrombus imaging and resonance fingerprinting, and how they will further aid in the rapid and accurate visualisation of stroke.

## The imaging of stroke

Neuroimaging is an important tool in the clinical diagnosis, management and treatment of stroke, and for determining prognosis (Caplan, 2006; Liebeskind, 2009). A few decades ago, diagnostic imaging revolved around the use of electroencephalography (EEG), thermography and radioisotope techniques. However, a breakthrough in imaging came with the introduction of CT and MRI, as both generate images of the human brain (Liebeskind, 2009). The main purpose of these neuroimaging techniques (Table 1) is to locate the affected vascular region of a stroke patient's brain, the infarct core (Box 2) and the penumbra (Box 2), as it is very important to know an infarct's location to minimise the severity of a stroke by using the most appropriate treatment.

Neuroimaging techniques are divided into two categories: structural and functional. The goal of structural imaging is to visualise the different anatomical structures of the brain and any deformities associated with it, such as a tumour, clot or bleeding, while the purpose of functional imaging is to assess activity in different parts of the brain. CT and MRI fall under the category of structural neuroimaging, whereas functional MRI (fMRI) and PET are different types of functional neuroimaging techniques.

### CT

**Fig. 1.**
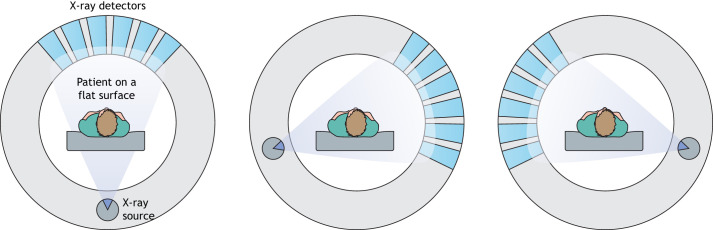
**Schematic of computed tomography (CT) scan.** During a CT scan, a patient lies on a flat surface, which passes through the scanner. The scanner consists of a rotating ring of X-ray sources and acquisition detectors. The acquisition system acquires X-rays from different angles of the brain and constructs the resulting cross-sectional, sliced images based on the tissue density calculated with the help of mathematical analysis (Sanelli et al., 2015).

CT is a very widely used neuroimaging technique because of its comparatively lower cost, shorter imaging time and wider availability compared to MRI. CT uses X-rays and detectors to produce cross-sectional images of the brain, as shown in Fig. 1. The contrast of the CT images depends on the differences in X-ray absorption between different tissue types (Sanelli et al., 2015). CT scans provide high-resolution images of osseous/bony structures rather than of soft tissues. Therefore, CT scans are generally recommended for the imaging of bone fractures.

### MRI

**Fig. 2.**
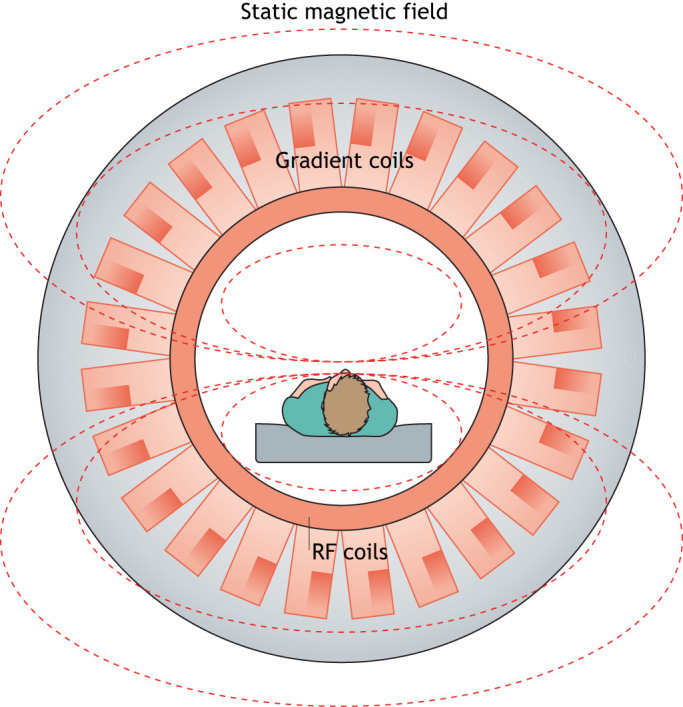
**Schematic of magnetic resonance (MR) scan.** A magnetic field generated by a magnet (grey) from the MR scanner aligns hydrogen nuclei (protons), which are usually randomly oriented, along its direction. This alignment results in the longitudinal magnetisation of the tissue. A radio frequency (RF) coil produces a brief signal (90° to the magnetic field) to flip the aligned spin of the protons, resulting in transverse magnetisation. When the RF signal is turned off, the spins of the protons relax back to their original states to realign with the static magnetic field. The longitudinal magnetisation returns to its original value and transverse magnetisation decays. During this relaxation process, the protons emit energy at the same RF, which is detected by the receiver coil to generate an image. The gradient coil assists in generating variations in the static magnetic field and the direction of the gradient (Zhang et al., 2016; Rastogi et al., 2015).

MRI is a multimodal imaging technology (as shown in Fig. 2) that is used to study anatomy and function by generating high-resolution images of the body's soft tissues, including the brain. It also provides better contrast between different tissues than CT. MRI uses the magnetic properties of hydrogen nuclei from water molecules in the tissues to visualise the internal structure of an anatomical region. In neuroimaging, different MRI sequences (Box 2) are used to visualise specific regions of the brain.

### Comparison of CT and MRI in stroke imaging

CT is widely used to initially evaluate patients with acute stroke symptoms within 24 h of stroke onset. In CT-generated images, blood products appear as distinct hyperintense lesions, which can be identified in the hyperacute phase (between 0 and 6 h) of haemorrhagic stroke. However, it is quite difficult to detect the hypointense lesion, a primary indication of ischaemic stroke, with CT images in the first few hours of a stroke. By comparison, MRI generates high-resolution images that outline the presence, size and location of a hyperacute cerebral ischaemic stroke (Heit et al., 2017; Kidwell et al., 2004; Jeena and Kumar, 2013).

**Fig. 3.**
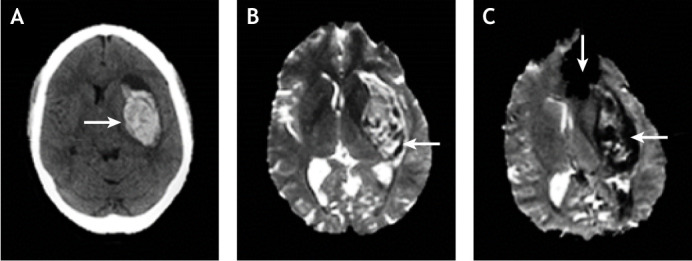
**CT and MRI images showing intracerebral haemorrhage.** (A-C) Images generated from CT (A), T2-weighted MRI (B) and GRE-MRI (C) scans, showing an intracerebral haemorrhage (horizontal arrows) at 2 h (CT) and 4.5 h (MRI) from symptom onset in an adult human. The vertical arrow in the GRE-MRI scan depicts the artefact caused by magnetic field inhomogeneity at the CSF/bone/air interface. Images reproduced with permission from Butcher and Davis (2009). These images are not published under the terms of the CC-BY license of this article. For permission to reuse, please see Butcher and Davis (2009). CSF, cerebrospinal fluid; CT, computed tomography; GRE, gradient recalled echo; MRI, magnetic resonance imaging.

Studies by Kidwell et al. (2004) and Fiebach et al. (2004) suggest that gradient recalled echo (GRE)-MRI, a multimodal magnetic resonance technique, produces equivalent results to a CT scan in the detection of hyperacute haemorrhage (Kidwell et al., 2004; Fiebach et al., 2004). Fig. 3 depicts the images of an acute haemorrhage generated from a CT scan, T2-weighted MRI scan and a GRE-MRI sequence scan (Butcher and Davis, 2009). One disadvantage of GRE images is that they are sensitive to magnetic field inhomogeneity, which reduces the signal intensity and generates artefacts, as shown in Fig. 3 (Butcher and Davis, 2009; Chavhan et al., 2009). Also, haemorrhages that appear acute in CT-generated images may appear as chronic in MRI-generated images, which makes CT a suitable technique for the initial diagnosis of acute stroke patients. These studies have also revealed that the MRI technique is superior to CT in detecting chronic haemorrhages and cerebral microbleeds (Haller et al., 2018). However, MRI is prone to artefacts caused by body movements due to longer scanning time (Butcher and Davis, 2009).

### CT approaches used to image stroke

**Fig. 4.**
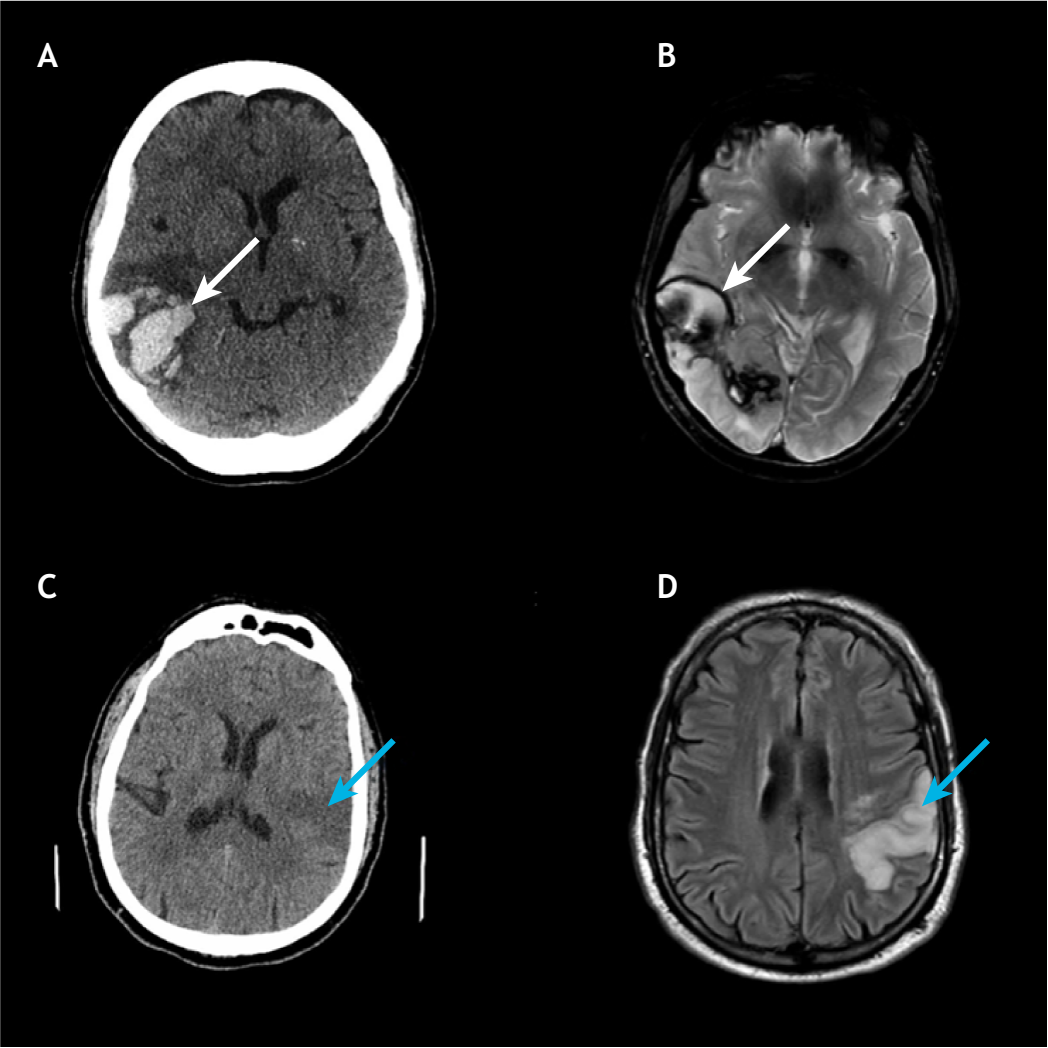
**CT versus MRI scans for detecting stroke.** (A,B) A haemorrhage can be seen clearly in the CT scan of a patient (A; white arrow), whereas it is less evident in an MRI scan (B; white arrow). (C,D) Whereas an ischaemic infarct is only faintly visible in the CT scan of a patient (C; blue arrow), it is clearly visible in an MRI scan (D; blue arrow). Images reproduced from Heit et al. (2017) and Rajdev et al. (2020) under the terms of the CC-BY 4.0 license. CT, computed tomography; MRI, magnetic resonance imaging.

In stroke imaging, CT is a mainstay in the initial evaluation of acute stroke patients as it can easily and rapidly visualise intracranial haemorrhages (as shown in Fig. 4). In current clinical practice, dynamic contrast-enhanced (DCE)-CT is also used to enhance image resolution and to assess the microvasculature of intracranial structures and other organs. In DCE-CT imaging, an iodine-based contrast agent is injected into the patient's body intravenously, which affects the measured X-ray absorption in tissues, leading to contrast enhancement in the generated images (O'Connor et al., 2011). CTA and CTP are two different modalities that are based on the DCE-CT imaging technique. CTA is a diagnostic tool that is used to assess intracranial structures and blood vessels to evaluate their occlusion or rupture, while CTP helps to quantify the potentially retrievable region, called the ‘penumbra’, and the unsalvageable ‘infarct core’ of an ischaemic stroke patient (Zhang et al., 2016; Wing and Markus, 2019). A study at the University of Cambridge, UK has also revealed that CTP performed at the time of initial imaging with CT has been quite successful in selecting patients for reperfusion therapy (Box 2) (Wing and Markus, 2019).

CT neuroimaging has several advantages over MRI, such as fast data acquisition, ease of access, interpretation and ease of imaging critically ill, claustrophobic or agitated patients, as well as excellent sensitivity for detecting intracranial haemorrhage (Box 1). However, CT also has its limitations, which are associated with the risk of exposure to ionising X-ray radiation and allergic reactions to contrast agents, especially in patients suffering from diabetes and renal disease (Zhang et al., 2016).

### MRI approaches used to image stroke

Technological advancements in MRI have improved stroke visualisation. Different MRI sequences can be used to obtain images and to alter the contrast between soft tissues. Below, we discuss how different MRI sequences are used to diagnose stroke.

T1- and T2-weighted image sequences (Box 2) are the conventional sequences that form part of all MRI protocols and enable tissue status to be visualised with greater detail than with CT. In T1-weighted images, tissues that contain water or fluid look dark, whereas fatty tissues look bright. In T2-weighted images, water and fluid-containing tissues look bright, whereas fat-containing tissues look dark (Sanelli et al., 2015; Zhang et al., 2016). T1-weighted images are suitable for the detailed visualisation of anatomy, while T2-weighted images help to detect tumours, oedema, infarction, or inflammation.

GRE (T2*-weighted) sequence (Box 2) and susceptibility-weighted imaging (SWI; Box 2) can both detect intracranial haemorrhages and cerebral microbleeds; however, SWI is more sensitive at detecting haemorrhages than GRE (Walsh et al., 2021; Cheng et al., 2013; Löbel et al., 2010; Copenhaver et al., 2009). This is due to its ability to distinguish between haemorrhagic and calcified matter (Chavhan et al., 2009; Mittal et al., 2009). However, a key limitation of SWI is that it has a long acquisition time, which can lead to artefacts due to movements caused by the discomfort of patients (Di Ieva et al., 2015).

Fluid-attenuated inversion recovery (FLAIR) sequences (Box 2) are highly sensitive for detecting multiple sclerosis plaques and subarachnoid haemorrhages (Lummel et al., 2011). One limitation of FLAIR scans is that they have a low signal-to-noise ratio, which decreases the contrast between the grey and white matter of the brain. However, a study by Zwanenburg et al. (2010) has reported that FLAIR scans at higher magnetic fields (7 Tesla; Box 2) have a high signal-to-noise ratio compared to scans at lower magnetic fields (1.5 and 3 Tesla). Nevertheless, image inhomogeneity remained a limitation, as reported in this study (Zwanenburg et al., 2010). This shortcoming led to the advent of three-dimensional (3D) FLAIR, which generates high-resolution images with a high signal-to-noise ratio (Tawfik and Kamr, 2020).

DWI is a very sensitive technique for the detection of acute ischaemic stroke (Nagaraja, 2021; Rastogi et al., 2015). It was first used in 1990 in feline models and was found to detect the infarct core within 45 min of stroke onset (Moseley et al., 1990; Rastogi et al., 2015). Perfusion-weighted imaging (PWI; Box 2) and MRA are DCE-MRI techniques in which a gadolinium (Gd)-based contrast agent (GBCA) is injected into the patient's body. PWI helps to visualise the cerebral ischaemic penumbra (Demeestere et al., 2020), while MRA aids in the detection of atherosclerosis, venous thrombosis and carotid artery injury in the neck and head (Birenbaum et al., 2011). Sometimes, DWI is unable to detect small infarcts and detects reversible lesions as irreversible. Thus, DWI is often used together with PWI to improve the visualisation of ischaemic stroke through the evaluation of DWI/PWI mismatches (Box 2) (Brunser et al., 2013; Edlow et al., 2017; Okorie et al., 2015; Blondin et al., 2009). DCE-MRI techniques can also demonstrate changes in blood-brain barrier permeability, which is significant in assessing the haemorrhagic transformation (Box 2), a complication of reperfusion therapy (Arba et al., 2020; Varatharaj et al., 2019; Li et al., 2018).

In addition to these well-established techniques, a new MRI modality has recently emerged called amide proton transfer (APT), which is based on the chemical exchange-dependent saturation transfer (CEST; Box 2) mechanism. APT can detect the endogenous protein and peptide concentration or pH changes in the stroke-affected tissues. This technique enhances the image contrast without any requirement for a contrast agent and can provide better information about tissue acidification in acute stroke. Tissue acidosis has been identified as a metabolic biomarker for pH changes in the ischaemic stroke penumbra. APT is used in conjunction with DWI and PWI to augment tissue classification (Lin et al., 2018; Ma et al., 2017; Zhou, 2011; Sun, 2020; Guo et al., 2016).

Apart from diagnosing stroke, MRI is also helpful in assessing the prognosis of stroke patients through fMRI. fMRI measures neuronal activity by assessing blood flow in the brain using the blood oxygen level-dependent contrast method discovered by Seiji Ogawa in 1990 (Ogawa et al., 2000). This specialised imaging technique exploits the correlation between neuronal activation and cerebral blood flow and is used to study the functional anatomy of the brain and to map brain activity. It can also provide insight into how the brain recuperates its lost functions following stroke or trauma (Crofts et al., 2020; Sanelli et al., 2015; Lindquist and Wager, 2015; Sutton et al., 2009).

Although MRI is more efficient than CT at detecting cerebral ischaemic stroke (Fig. 4), it has its limitations, such as higher costs, longer scanning times and a noisy, tunnel-like structure that can make it challenging to scan critically ill, claustrophobic or agitated patients, or patients that have pacemakers, aneurysm clips or other metallic objects inside their bodies. Nevertheless, the physiological information obtained from MRI scans can significantly assist in the clinical management of stroke patients by providing accurate information on the size of the infarct core, the penumbra and the site of occlusion (González, 2012; Provost et al., 2019; Bang et al., 2018; Kim et al., 2014). For example, in a multicentre clinical trial, the intravenous administration of the thrombolytic drug recombinant tissue plasminogen activator (rtPA; Box 2) 3 h after stroke reduced infarct growth based on DWI/PWI mismatch imaging (Albers et al., 2006; Davis et al., 2008; Bang et al., 2018). However, large-scale clinical trials are still needed to demonstrate the value of MRI for guiding precise and accurate treatment of stroke patients (González, 2012).

## Advances in the neuroimaging of stroke

Neuroimaging has made a considerable contribution to the diagnosis of stroke and has also advanced our understanding of the brain's structural and functional anatomy. In the past few decades, various technological improvements, such as the implementation of different pulse sequences and the use of intravenous contrast agents, have advanced the uses of neuroimaging in medical diagnostics. Here, we discuss technological advancements that are still in development.

### Ultra-high-field MR

MR scanners with an ultra-high magnetic field (UHF), ≥7 Tesla, have recently been developed. These scanners can produce a high signal-to-noise ratio, which reduces scanning time and provides higher spatial resolution in the resulting images relative to conventional low-magnetic field MR scans. UHF-MR scanners can also visualise microscopic anatomical details that cannot be detected with low-field MR scanners (Barisano et al., 2019; Balchandani and Naidich, 2015; Kathiravan and Kanakaraj, 2013; Retico, 2018). UHF-MR scanners also have great sensitivity for low-γ nuclei (^23^Na, ^31^P), which are less abundant than protons in human tissues. Enhanced sensitivity of UHF-MR scanners to these nuclei helps in the assessment of changes in phosphate metabolism, intra- and extracellular sodium levels linked to ischaemic stroke, brain tumours, Alzheimer's disease and other neurological disorders. They thus provide critical information related to intracellular pH, metabolic pathways and neuroinflammation, which cannot be obtained using low-field MR scanners (Qiao et al., 2006; Huhn et al., 2019; Stovell et al., 2017; Wijnen et al., 2012; Komoroski et al., 2011; Biller et al., 2016). Although UHF-MR scanning provides metabolic information, its clinical application, which could range from diagnosis to drug development for stroke, has yet to be realised (Platt et al., 2021).

Despite the improved imaging capabilities of UHF-MR, it is not without limitations. Magnetic field inhomogeneity, increased specific absorption rate (SAR; Box 2) and the need for specialised hardware and software solutions to remove artefacts are some of the technological challenges associated with the use of UHF-MR scanners (Retico, 2018; Balchandani and Naidich, 2015; Ladd et al., 2018). However, research is underway to mitigate the issues of inhomogeneity by adjusting the magnitude and phases of radiofrequency (RF) pulses in a region of interest (RF field shimming; Box 2) and by using multitransmit RF coils for parallel transmission (Deniz, 2019; Stara et al., 2017; Mao et al., 2006). The use of metamaterials, which are artificial composite materials that can interact with electromagnetic radiation in a desired manner, in MRI is also under investigation to control SAR and to enhance UHF-MR performance (Schmidt et al., 2017; Duan et al., 2019). Although UHF-MR scanners are more sensitive than are conventional MR scanners, there is still a long way to go before these scanners can be used in a clinical setting.

### MR fingerprinting

Although MR imaging has revolutionised the field of medical diagnostics and imaging, it is non-quantitative in nature, which is a key limitation. Current scanners produce qualitatively weighted images, i.e. contrast is weighted using physical parameters, such as longitudinal relaxation time (T1) and transverse relaxation time (T2). The description of MR images as being hyperintense or hypointense generally provides relative information rather than absolute values of physical parameters (Retico, 2018; Panda et al., 2017). MR fingerprinting (MRF) is a major step towards addressing this limitation by providing quantitative MR imaging (Hsieh and Svalbe, 2020; Ma et al., 2013) of various tissue properties, such as T1 and T2, diffusion and perfusion, simultaneously. By comparison, current MRI techniques can only assess a few such properties at a time. MRF provides an alternative method for analysing the complex physical changes within a tissue that can be helpful in the early detection of disease. It can also be used to identify a specific target tissue, leading to an increase in the specificity and sensitivity of MR-mediated diagnostics (Ma et al., 2013; Panda et al., 2017). According to one study, MRF can identify different states of ischaemic stroke, can quantitatively assess the white matter, and can help infer the physical and physiological characteristics of an infarct region (Ma et al., 2014). In another study, MRF was used to analyse microvascular properties such as cerebral blood volume and mean vessel radius (Christen et al., 2014). Small-scale clinical trials have shown that MRF-generated quantitative maps can differentiate between diseased and healthy brain tissue. However, large-scale clinical trials are still needed to validate MRF for clinical implementation (Christen et al., 2014; Hsieh and Svalbe, 2020; Su et al., 2017). The main limitation of this technique is long computational time, which needs to be overcome through the processing of image data by artificial neural networks (Oksuz et al., 2019; Hoppe et al., 2019).

### Neural network mapping

Machine learning or artificial neural networks (ANNs) (Lundervold and Lundervold, 2019) are algorithms that make a computer capable of solving complicated problems from a large data set. The development of convolutional neural networks (CNNs), a class of ANNs used for the analysis of images, has also triggered a breakthrough in the field of medical imaging, diagnostics and data analysis. CNNs have shown outstanding performance in the analysis of medical images, such as in lesion segmentation, anatomical segmentation and classification (Lundervold and Lundervold, 2019; Bernal et al., 2019). This approach can also remove the artefacts generated by magnetic resonance spectroscopy, which arise due to several sources, such as magnetic field inhomogeneity, subject movement, reduced peak-to-noise ratio, and improper suppression of water and lipid signals (Gurbani et al., 2018). CNNs automatically detect and filter out the poor-quality spectra and artefacts with high sensitivity and specificity. Recently, Arab et al. (2020) developed a fully automatic machine-learning algorithm to quantify haemorrhage volume using CT-generated images from 55 stroke patients. This fully automated analysis of stroke data, with an assessment time of 0.7 s for a whole-head image, exhibited better reliability than Primary Intracranial Haemorrhage Probability Estimation using Random Forests on CT (PItcHPERFeCT), a commonly used machine-learning model for CT-image analysis of stroke, which takes ∼1412 s (Muschelli et al., 2017; Arab et al., 2020). In addition to processing and analysing images, ANNs can potentially aid in the management of stroke patients. A recent study of health survey data from 15,099 stroke patients reported that an ANN model could predict the risk of stroke with an accuracy of 83.48% (Cheon et al., 2019). Another study by Yu et al. (2020) analysed ischaemic lesion data from the initial MRI of 182 stroke patients using a machine learning model, which could predict the infarct lesion prior to medical intervention in 77% of patients. However, this study was carried out using specific MRI techniques at different institutions using different MRI scanners; thus, standardisation of the results is difficult. Moreover, this study collected data from patients exhibiting ischaemic stroke symptoms within 24 h of the onset of stroke. The lesions might not be fully evolved within this timeframe, causing error in the prediction of stroke.

The Alberta Stroke Program Early Computed Tomography Score (ASPECTS; Box 2) is a widely used score to assess ischaemic changes in the treatment of acute stroke. In particular, it helps with the selection of patients suitable for endovascular therapy (EVT; Box 2). Recently, machine learning-based algorithms have been used to calculate this score (Bang et al., 2018). For example, a multicentre trial reported that ASPECTS calculated by machine learning-based algorithms like the e-ASPECTS software were similar to those determined by neuroradiologists (Nagel et al., 2017). Based on clinical trials, machine learning with neural networks can also help to predict stroke and its risk factors, to differentiate between stroke and stroke mimics (Box 2), to select ischaemic stroke patients for reperfusion therapy and to predict the outcomes of reperfusion therapy (Asadi et al., 2014; Weng et al., 2017; Abedi et al., 2017).

Despite its advantages, machine learning has limitations such as hardware dependency, the need for highly skilled people and for large, organised data sets to train algorithms on, and difficulty with data interpretation (Soun et al., 2021). Although several machine learning software platforms are available that contain algorithms with which to analyse different aspects of stroke, the use of these platforms in a clinical setting is still at an early stage (Soun et al., 2021).

## Clinical improvements supported by MRI advances

Conventionally, MRI has been used as a second line of diagnosis after CT, if detailed information about damage due to stroke is required. Recent multicentre trials such as Diffusion-Weighted Imaging Evaluation for Understanding Stroke Evolution Study 2 (DEFUSE-2) and General or Local Anaesthesia in Intra-arterial Therapy (GOLIATH) have demonstrated the potential of MRI in the diagnosis and treatment of stroke patients (Lansberg et al., 2012; Simonsen et al., 2018). In the following section, we discuss the clinical impact of MRI in the management of stroke.

### MRI and reperfusion therapy

Reperfusion/revascularisation therapy is the main treatment for patients with acute ischaemic stroke and entails treating stroke patients with rtPA and EVT (The National Institute of Neurological Disorders and Stroke rt-PA Stroke Study Group, 1995; Bang et al., 2018). However, a considerable number of ischaemic stroke patients are ineligible for reperfusion therapy (Molina, 2010; Chia et al., 2016) because of their age, clot location, stroke severity, extent of the ischaemic penumbra and the pattern of collateral flow, which can all lead to the failure of reperfusion treatment (Molina, 2010).

Several research reports have indicated that multimodal neuroimaging techniques may be beneficial in selecting acute ischaemic stroke patients for reperfusion therapy (Molina, 2010; Chia et al., 2016; Bang et al., 2018). Recent clinical trials have indicated that the use of MRI can select patients for EVT rapidly, efficiently and within a similar timeframe as that achieved when using CT (Simonsen et al., 2014, 2018). The risk of haemorrhagic transformation increases if acute ischaemic stroke patients are treated with rtPA within ≤4.5 h from the onset of stroke (Dela Peña et al., 2017). A quality improvement programme launched by the American Stroke Association (ASA) promotes reducing door-to-needle time for rtPA administration to ≤60 min for better management of acute stroke patients (Fonarow et al., 2011). A study by Shah et al. (2015) has demonstrated that screening acute stroke patients with a multimodal MRI-based approach (SMART) can help to treat acute stroke with rtPA within the time window advised by the ASA (Shah et al., 2015). In addition, a single-institution study of 62 stroke patients found that a fast MRI protocol that includes DWI, FLAIR, GRE, PWI [also known as magnetic resonance perfusion (MRP)] and MRA can be accomplished in ∼6 min, which is faster than a comprehensive acute stroke CT protocol (Nael et al., 2014). Moreover, automated software, such as Rapid Processing of Perfusion and Diffusion (RAPID), is currently being used to enable the fast post-processing of PWI and DWI data for further clinical assessment. This software provides almost immediate (∼5 min) information on the location and extent of the infarct core and penumbra through DWI/PWI mismatch, which can inform the selection of acute ischaemic stroke patients for reperfusion therapy (Vyas et al., 2019; Straka et al., 2010). Despite its benefits, however, this software is not without limitations, such as motion artefacts caused by patient movement and high cost due to the use of additional sequences, which both hinder the software's widespread use in clinical settings (Vyas et al., 2019).

### MRI enhancement methods

MRI enhancement methods improve the visibility of physical and physiological abnormalities in the scans of body tissues.

#### GBCAs

**Fig. 5.**
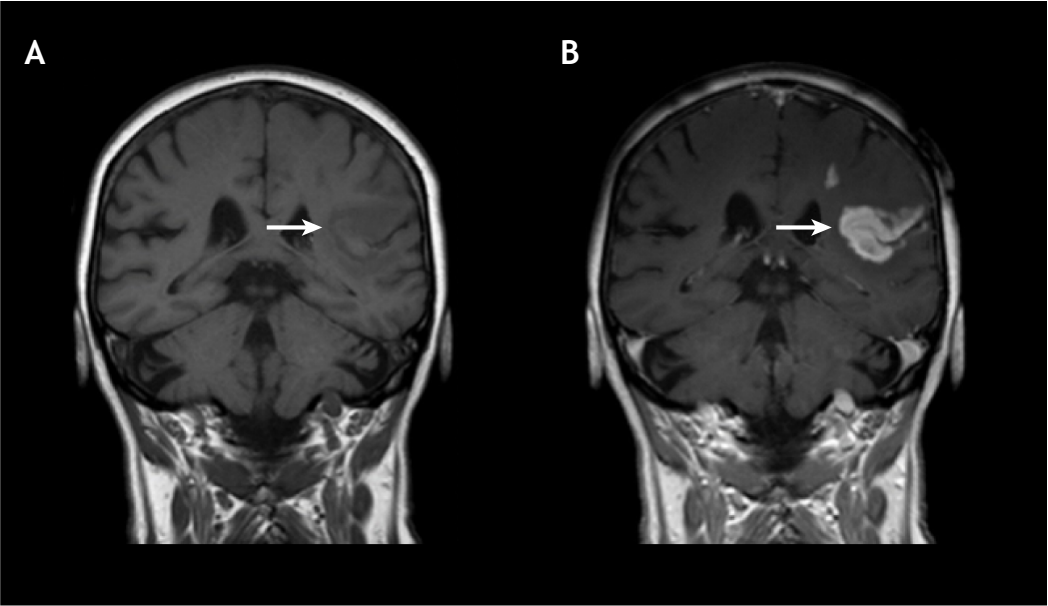
**Effect of contrast agent.** (A,B) T1-weighted MRI of an adult brain showing infarction (white arrows) without contrast agent (A) and with contrast agent (B). Images reproduced with permission from Friebe (2016). These images are not published under the terms of the CC-BY license of this article. For permission to reuse, please see Friebe (2016).

Clinically available GBCAs are mainly employed as extracellular fluid agents in ligand form that pass through the patient's body without interacting with their cells. Although GBCAs provide excellent contrast between different anatomical structures, they do so in a non-specific manner. They are currently used in 60% of neurological MRI scans to enhance the contrast in MR images (Wahsner et al., 2018). GBCAs are categorised as paramagnetic contrast agents that shorten the T1 and T2 relaxation time of protons in their vicinity, leading to enhanced contrast in the resulting images (as shown in Fig. 5). These contrast agents help to detect stroke, blood-brain barrier disruption, unhealthy tissues, tumours and other neurological manifestations (Xiao et al., 2018). In addition, the effect of GBCAs is immediate in comparison to the radioactive tracers used in nuclear medicine, as the GBCA can be administered while a patient is in the scanner (Wahsner et al., 2018; Khairnar et al., 2019). According to one study, their rate of adverse effects is also very low (Prince et al., 2011). However, two other studies have reported that GBCAs can cause a fatal condition known as nephrogenic system fibrosis (Marckmann et al., 2006; Grobner, 2006). As such, research is currently underway to find alternatives to GBCAs, such as CEST, nanoparticle-based and direct detection agents, to use as new MRI contrast agents and to investigate their potential applications in stroke diagnosis, as we discuss further in this section (Walker-Samuel et al., 2013; Ruiz-Cabello et al., 2011; Ward and Balaban, 2000; Pellico et al., 2019).

#### CEST contrast agents

CEST contrast agents are molecules with exchangeable protons such as OH and NH. When irradiated with an RF pulse, their magnetisation is transferred to bulk water molecules through an exchange of free protons. This transference process reduces the bulk water signal and generates the resulting MRI contrast (Xiao et al., 2018; Jones et al., 2018). In a pilot clinical trial of 12 ischaemic patients analysed retrospectively, the contrast-to-noise ratio of the ischaemic core was investigated using quantitative APT-based CEST imaging (Msayib et al., 2019), which was found to produce a higher contrast-to-noise when imaging the ischaemic core. This may give further insight into metabolic pathways and pathophysiology of stroke (discussed in the ‘MRI approaches used to image stroke’ section). However, further clinical trials on the modelling of APT data are required to realise the benefit of CEST imaging.

#### Nanoparticle-based agents

Nanoparticle-based agents have also been investigated for MRI enhancement (Pellico et al., 2019). For example, supramolecular amorphous-like iron oxide (SAIO) nanoparticles have been developed for the 3D mapping of vascular structures in the brain. The smaller size (∼5-50 nm) enables them to cross the blood-brain barrier and to be easily excreted from the body. SAIOs show very good biocompatibility and imaging performance when tested against a GBCA in a rodent MRI model (Shin et al., 2021).

#### Direct detection agents

Direct detection agents, such as perfluorocarbons (PFCs), are inert organic molecules in which hydrogen is replaced with fluorine (Xiao et al., 2018). PFCs act as oxygen carriers and, when combined with hyperoxia, can help to identify ischaemic penumbra through continuous changes caused by tissue metabolism. PFCs have been proposed to enhance the imaging of penumbra using Glasgow oxygen level-dependent (GOLD) MRI in the rat stroke model (Deuchar et al., 2018).

#### Targeted contrast agents

Recently, a new class of MRI contrast agents has been developed to detect molecular biomarkers like proteins, ions or metabolites, and to increase our ability to use MRI to assess pathological tissue (Wahsner et al., 2018; Sinharay and Pagel, 2016; Lux and Sherry, 2018; Louie and Meade, 2000). They come in two main forms: activatable and biochemically specific targeted agents.

Activatable contrast agents work by producing a signal that is altered in response to specific external stimuli such as pH, light, enzymatic activity, metal ions and temperature change (Wahsner et al., 2018; Tu et al., 2011). Biochemically specific contrast agents involve conjugating an extracellular fluid (ECF) contrast agent such as GBCA with a targeting vector that localises the agent to a specific protein or target organ to provide local contrast enhancement. Although there are safety concerns regarding the use of GBCAs, using them with a targeting vector might help in concentrating GBCAs to the target site rather than allowing non-specific absorption in the body (Wahsner et al., 2018).

Thrombosis is a major cause of stroke, and acute thrombosis detection through targeted MRI imaging could lead to better clinical decision making (Vymazal et al., 2009; Dutra et al., 2019). In one study, fibrin-targeted Gd (III) contrast agents were used to detect carotid thrombosis induced in guinea pigs (Sirol et al., 2005). The use of the fibrin-targeted contrast agent increased thrombus detection by MRI to 100% in comparison to the 41.6% achieved when using non-specific Gd MRI (Sirol et al., 2005; Vymazal et al., 2009; Overoye-Chan et al., 2008). Thus, the detection of molecular biomarkers through the use of such contrast agents could enhance MRI and thus improve clinical decision making. However, the identification of a biomarker that has high specificity and affinity for its target, without any complications, poses a key challenge in this difficult, yet innovative, approach (Wahsner et al., 2018).

#### Arterial spin labelling

Currently, DCE-MRI and DCE-CT are the primary techniques used to measure cerebral blood perfusion with the help of contrast agents. However, there is a non-contrast MRI technique called arterial spin labelling (ASL) that uses water molecules in the blood as an endogenous tracer (Petcharunpaisan et al., 2010; van Osch et al., 2018). The use of an endogenous tracer enables patients with renal disease, those who are allergic to contrast agents or those who need regular follow-ups to be assessed using MR scans. According to retrospective analyses by Yoo et al. (2018b) (*n*=51) and Nedunchelian et al. (2021) (*n*=95), ASL could be used to inform treatment decisions for stroke patients and to predict the functional outcomes of ischaemic stroke patients after endovascular therapy (Yoo et al., 2018b; Nedunchelian et al., 2021). However, the use of this technique in place of conventional perfusion techniques awaits large-scale clinical trials (Nedunchelian et al., 2021).

### Thrombus imaging

Although MRI and CT can be used to detect blood vessel occlusion, they do so by revealing a blockage in blood flow rather than the thrombus itself. However, direct thrombus imaging using MRI and CT is an emerging technology that could improve the treatment of patients with acute ischaemic stroke by enabling the size and location of the thrombus to be directly visualised (Kim et al., 2016b). According to one study, plaque rupture in arteries is the major cause of ischaemic stroke (Virmani et al., 2002). Conventional imaging techniques cannot identify such high-risk plaques or thrombi. Without direct thrombus imaging, it is difficult to decide on an appropriate rtPA dose to give to an individual patient, as the recommended rtPA dose of 0.9 mg/kg (The National Institute of Neurological Disorders and Stroke rt-PA Stroke Study Group, 1995) can be too low for some patients, leading to a decreased rate of thrombolysis, and too high for others, with the concomitant increased risk of haemorrhagic transformation. The direct imaging of a thrombus could thus help to determine the dosage of intravenous rtPA by providing information on its size, distribution, location and nature (Kim et al., 2016b).

In 2003, a study by Moody et al. demonstrated that MR-based direct thrombus imaging can detect methaemoglobin, an indicator of plaques, in 84% of patients with ischaemic stroke (*n*=63) (Moody et al., 2003). CT-based imaging techniques have also been studied for their ability to directly detect thrombi. In a retrospective study of 35 patients with cerebral thromboembolism (Box 2), SWI-MRI thrombus imaging was superior to CT-based imaging in the diagnosis of acute cerebral infarction (Mamlouk et al., 2012). In addition, Kim et al. (2016a) showed that a fibrin-targeted nanoparticle could be used for direct CT-based imaging of cerebral thrombi in a mouse stroke model.

As discussed above, the main aim of thrombus imaging is to stratify stroke patients for EVT by identifying and characterising thrombi. Several studies have attempted to implement various thrombus parameters, such as permeability, volume and length, as well as scoring systems, such as the clot burden score, with which to characterise thrombi (Santos et al., 2016; Yoo et al., 2018a; Polito et al., 2017; Puetz et al., 2008). However, location is still the most widely used parameter to characterise a thrombus (Guerrero et al., 2019). Recently, a multicentre randomised clinical trial (MR CLEAN) evaluated the association of thrombus imaging characteristics, such as location, length, clot burden score and distance from the internal carotid artery terminus to the thrombus, with the functional outcome of EVT (Dutra et al., 2019). This study observed that distal occlusion, shorter clots and higher clot burden score predicted a better functional outcome in 408 ischaemic stroke patients. However, further studies are required to establish thrombus imaging as a routine practice to assess the functional outcomes of EVT for ischaemic patients (Guerrero et al., 2019).

## Conclusion

Neuroimaging is an indispensable diagnostic method. In diagnosing stroke, its greatest advantage is that it enables clinicians to rapidly identify patients most likely to benefit from certain treatments, including thrombolytic agents and surgical treatment. Owing to its wider availability and shorter scanning time, CT is still generally the preferred method used to initially evaluate acute stroke patients over non-ionising MRI. However, technological advances in MRI in recent years have strengthened its potential as the imaging platform in a routine clinical stroke protocol (MacIntosh et al., 2013; Burgess et al., 2011).

Multimodal MRI is an invaluable technique for initially diagnosing ischaemic stroke, and for determining the site and size of the infarct area, key information that can assist clinicians to determine appropriate interventions and to efficiently manage stroke patients. The use of MRI in the diagnosis and treatment of stroke includes the use of SWI to identify cerebral haemorrhage, PWI to determine the extent of the ischaemic penumbra, and both PWI and DWI to assess stroke. Moreover, recent innovations and advancements in MRI, such as the use of an ultra-high magnetic field, MRF and neural network mapping, are constantly being improved for use in the prediction and clinical management of stroke. These technological advances will also help to extract important information hidden in the avalanche of MR data gathered in clinical centres worldwide. In addition, the advent of novel MR contrast agents has revolutionised the field of modern diagnostic technologies. These agents provide essential anatomical information that cannot be obtained when using non-invasive methods, and research to develop more targeted MR contrast agents is currently underway. One expected advancement would be the integration of artificial intelligence and machine learning with efficient contrast agents to improve image quality and to reduce the data acquisition time, which are crucial for the accurate diagnosis and early treatment of stroke. Further improvements in MR scanning techniques are likely to come from the use of MRI in animal models, which have been used to explore the targeted delivery of contrast agents and to better understand brain function. Although MRI has the potential for diagnosis of stroke, it is still not the first-line imaging approach. Large-scale clinical studies are required to further develop these diagnostic approaches (Denic et al., 2011; Shen and Duong, 2016). Moreover, the technological challenges that come with MRI need to be addressed to better apply the full benefits of MRI technology in clinical settings.
